# Efficacy of traditional Indian diet (*Ayush ahara*) on muscle strength and Sarcopenia: A scoping review

**DOI:** 10.1016/j.jaim.2025.101265

**Published:** 2026-01-30

**Authors:** Rahul Katkar, Usha Rana, Sriloy Mohanty, Monika Pathania

**Affiliations:** a Department of AYUSH, All India Institute of Medical Sciences, Rishikesh, Uttarakhand, 249203, India; b Dept. of Shalyatantra, Uttarakhand Ayurveda University, Dehradun, Uttarakhand, 248001, India; c Dept. of Geriatric Medicine & Head of Department (AYUSH), All India Institute of Medical Sciences, Rishikesh, 249203, Uttarakhand, India

**Keywords:** Muscle strength, Body composition, Physical fitness, Sarcopenia, *Ayush ahara*

## Abstract

Sarcopenia is a degenerative musculoskeletal condition that affects older persons with the hallmark of loss of muscle mass and function. Modern management is limited to nutrition, exercise, and lifestyle modifications, with no approved drugs. Ayurveda recognizes a similar condition called *Mamsa kshaya* due to vitiation of *Vata dosha*, which reflects the clinical picture of sarcopenia, and offers *Ayush Ahara*, a therapeutic dietary approach.

To assess the clinical evidence for the role of traditional Indian diet (∼*Ayush ahara*) in improving muscle strength and managing sarcopenia.

A scoping review was conducted according to PRISMA-ScR. Literature published from 2015 to 2025 was searched across various databases. Inclusion criteria: randomized controlled trials involving human subjects assessing the effect of traditional Indian diet on muscle strength-related outcomes. Non-English, non-full-text, in vitro/in vivo studies, and reviews were excluded.

The nine studies were included. *Ayush ahara,* such as green gram, dried grapes, turmeric, cow milk, finger millet, spinach, and almonds, was associated with improved muscle strength, physical performance, reduced inflammation, and enhanced bone mineral density.

*Ayush ahara* shows potential for improving muscle strength and managing sarcopenia. The reviewed RCTs showed statistical improvements in muscle strength measures, indicating the need for targeted clinical trials to validate these preliminary findings. Support further exploration of Ayurveda-based nutritional strategies as a complementary approach to musculoskeletal aging.

## 1 Introduction

Sarcopenia is described by the Asian Working Group for Sarcopenia (AWGS) 2019 criteria as age-related loss of muscle mass, low muscle strength, and/or impaired physical performance [1]. In ICD-10, sarcopenia is classified under “M628 II Other specified disorder of muscle.” Sarcopenia affects 10.6 %–16 % of older people worldwide and 12.6 % –17.5 % of aged Indians [2]. Particularly for elderly people, muscle strength is essential for preserving functional independence, mobility, and general quality of life. Diet is one lifestyle component that can be modified to affect the health of bones and muscles. In addition to lowering the risk of metabolic syndrome and other serious chronic diseases, eating a healthy diet can increase longevity [3]. Consuming a diet high in nutrients and bioactive components seems to be essential for maintaining skeletal remodeling and delaying the loss of muscle and bone mass^.^ [4] Sarcopenia is related to higher risks of mortality, falls, disability, and functional muscle impairment. Several key mechanisms have been implicated in the pathogenesis of sarcopenia, including mitochondrial dysfunction, chronic inflammation, oxidative stress, and hormonal changes. These processes lead to impaired muscle protein synthesis, increased proteolysis, and reduced regenerative capacity of skeletal muscle [5]. There are currently no approved medicines to treat sarcopenia; instead, lifestyle changes, physical therapy, and nutritional interventions are the main methods of management [6]. Growing interest is directed toward traditional dietary practices, particularly those rooted in Ayurveda.

There is a physiological increase of *Vata dosha* in *Jara avastha* (∼old age) [7]. Due to this physiological and pathological vitiation of *Vata dosha* in old age, it reflects the clinical picture of *mamsa kshaya* [8,9] leading to sarcopenia. The visible and measurable impact, including reduced grip strength and gait speed, is localized to *Mamsa dhatu*. According to the *Dhatuparinama krama* (sequential transformation of *Dhatus*) described in classical texts, nourishment of *Mamsa dhatu* is dependent on the proper formation and quality of preceding *dhatus—Rasa* and *Rakta dhatu*. If *Mamsa dhatu is* depleted, *Meda dhatu* formation may also be compromised. The progressive decline in *Agni* (∼digestive fire) and weakened *Dhatu poshana* (∼tissue nourishment) contribute to muscle deterioration in elderly individuals [10]. *Ahara* is considered as one of the three pillars (∼*Trayo-upasthamba*) [11] and is the most excellent of all medicines [12]. The basic idea of Ayurveda's preventive and therapeutic practices is *Pathya ahara* (∼wholesome diet) and *Vihara* (∼wholesome lifestyle). The concepts of *Pathya* refer to *Ahara* and *Vihara*, which are beneficial to an individual's channels, constitution, and strength [13]. *Ayush Ahara*, comprising a traditional Indian diet as prescribed in Ayurveda, is believed to contribute to musculoskeletal strength support *Dhatu poshana* (∼tissue nourishment), improve *Bala* (∼strength), and enhance *Ojas* (∼vitality) [14]. Despite their longstanding use and cultural relevance, evidence regarding their efficacy in improving muscle strength remains scattered. In this scoping review, we discuss the traditional Indian diet (∼*Ayush ahara*), which is specific for improving muscle strength in sarcopenia.

### 1.1 Objectives and review questions

This scoping review's primary objective is to analyze and compile the available data regarding the effectiveness of the *Ayush ahara* in improving muscular strength. This review seeks to identify the types of traditional diets used, populations studied, outcomes assessed, and the methodological characteristics of the available evidence.

The following core question guides the review.

• What is the current scope of evidence regarding the role of *Ayush ahara* in enhancing muscle strength across different populations?

## 2 Methods

### 2.1 Protocol and registration

The scoping review protocol was registered on the Open Science Framework on May 13, 2025 with the link osf.io/h9bav/.

## 3 Eligibility criteria

**Inclusion Criteria:** (1) Randomized clinical trials involved human participants with intervention as the traditional Indian diet (∼*Ayush ahara*) on improving muscle strength. (2) Original research papers published between the years 2015 and 2025 (3) English, free full-text articles.

**Exclusion Criteria:** (1) Randomized clinical trials involved human participants with intervention as the only diet but not the traditional Indian diet (∼*Ayush ahara*) for improving muscle strength. (2) studies that used intervention as pharmaceutical drugs or Ayurveda drugs. (3) Other study designs: In vivo/in vitro studies, observational studies, case reports, case series, systematic reviews, or other review types. (4) conference proceedings or abstracts.

### 3.1 Data source

A comprehensive search of the literature published in various database was conducted to identify articles on the efficacy of the *Ayush ahara* on muscle strength.

### 3.2 Electronic search and selection of the source of evidence

The search strategy was created by the researcher and then refined upon by group discussion. Team members removed duplicate keywords from the final search strategy. The search strategy was used to identify relevant studies in the PubMed, Scopus, and Embase databases and providing total search results in supplementary file 1. The search was conducted on [May 1, 2025]. Initially, 3048 records were found across the PubMed, Scopus, and Embase databases. All retrieved citations were imported into Rayyan software (https://new.rayyan.ai/) for screening and selection. Out of this, 289 duplicate records were removed. After this deduplication step, two reviewers independently screened the titles and abstracts of a total of 2759 articles.

**Fig. 1:**
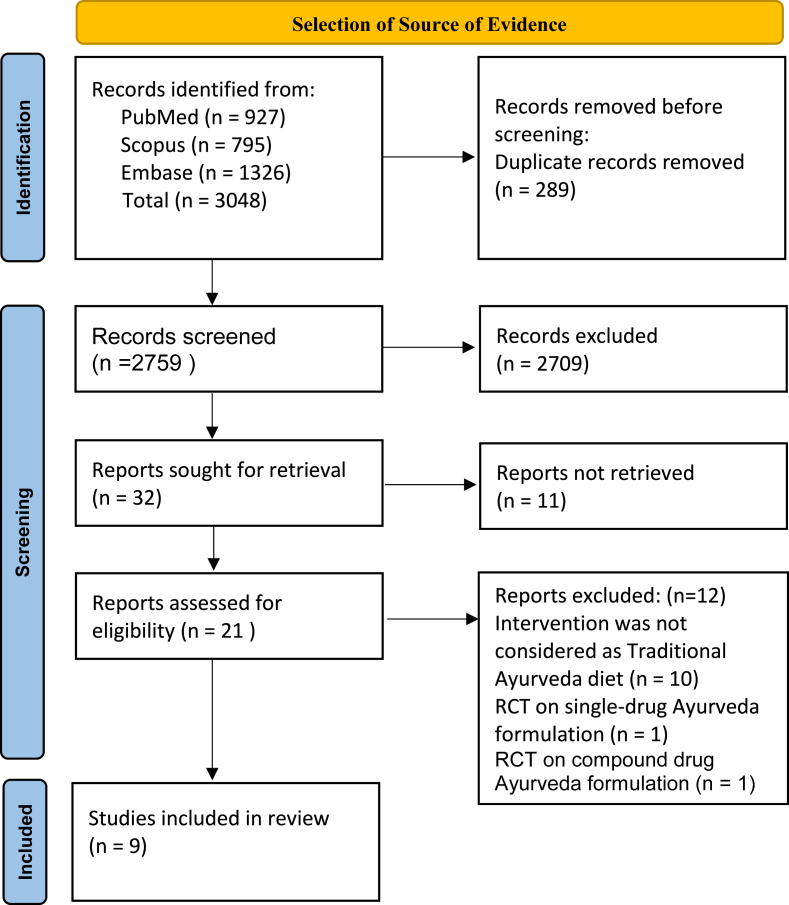
PRISMA Flow diagram

Out of these 32 records are sought for retrieval based on the application of the following filters: Interventions considered as diet, Publication date—last 10 years (from year 2015–2025); Article type—Randomized clinical trial (RCT). After this step, 21 full-text articles were assessed against the eligibility criteria, and 9 full-text articles met the predefined eligibility criteria. The 12 articles were excluded in the final analysis due to reasons like the intervention not being considered a traditional Ayurveda diet (n = 11), an RCT on a single Ayurveda formulation (n = 1), an RCT on a compound Ayurveda formulation (n = 1), and the publications not being taken into consideration (See PRISMA flow diagram—Fig. 1.). The 9 research articles were assessed by all authors, which fulfills the pre-defined eligibility.

### 3.3 Data charting process

Two reviewers abstracted data on selected articles, such as first author, year of publication, country of origin, sample size, dropout, age-gender distribution, type of study design, intervention details, co-intervention details, study duration, measurement tools, outcome, evaluation sessions, and adverse events. After charting the data individually, the reviewers discussed the results. (see Table 1).

**Table 1:** Synthesis of related papers.

Sr. N	First author and year	Country of origin	N	Sex & Age Distribution	Study design	Intervention	Co-Intervention (Type of exercise)	Dropout	Duration	Measurement Tools	Outcomes	Evaluation session	Adverse Events
1	Bartholomae E et al., 2019	USA	37	100 % vegetarian adults (sex not specified); 18–55 years old	RCT	Mung bean protein (18g/day) vs control biscuit (4g/day)	–	12	8 week	Grip dynamometer, DEXA	Grip, flexor & extensor strength, lean mass	Baseline & week 8	None reported
2	d'Unienville NMA et al., 2019	Australia	96	Male endurance athletes (18–60 years.)	Single-blind RCT	Almond-grape-cranberry mix vs oat snack	Endurance training (athlets)	Not specified	5 Weeks	5-min time trial, rHRI, oxidative stress markers	Performance, NO synthesis, muscle recovery	Pre- & post-training	None reported
3	Salehi M et al., 2021	Iran	80	Females (20–30 yrs)	Double-blind RCT	Curcumin 500 mg/day vs placebo	Moderate exercise (walking/treadmill)	Not specified	8-week	VO_2_ max, CRP, LDH, MDA	Inflammation, aerobic capacity	Pre & post-intervention	None reported
4	Ayubi et al. (2023)	Indonesia	24	Male, 18–22 years	RCT	Curcumin supplementation	Resistance training (3 × /week)	Not specified	8 weeks	CK, LDH, muscle soreness scale	Muscle damage indicators, recovery	Pre- and post-intervention	Not reported
5	Chiang FY et al., 2021	Taiwan	35	Elderly (mean 84.9 ± 6.1 yrs), both sexes	RCT	Milk (400 mL/day) + resistance training	Resistance training	Not specified	12 weeks	Handgrip strength, calf circumference	Strength, mass	Baseline, every 4 weeks	None reported
6	Granic et al. (2020)	United Kingdom	30	Male & Female, ≥65 years	Pilot RCT	Milk post-exercise	Resistance training (2 × /weeK)	4	6 weeks	Handgrip strength, SPPB, PA questionnaire	Feasibility, strength, function	Baseline & follow-up (6 weeks)	Mild soreness, no serious events
7	Sahaya Rani G et al., 2021	India	150	Premenopausal women	RCT, parallel arms	Finger millet laddu (3 × /week) + daily exercise	Structured exercise (unspecified)	Not specified	12 weeks	BMD, serum Ca/P, ALP	Bone density, serum markers	Baseline & post-intervention	None reported
8	Perez-Pinero S et al., 2021	Spain	45	Adults >50 yrs, both sexes	Double-blind RCT	Spinach extract (2 g/day) + resistance training	Moderate exercise (walking/treadmill)	Not specified	12-Week	DEXA, isokinetic strength test	Muscle mass, isometric & isokinetic strength	Pre & post	None reported
9	Rayo VU et al., 2024	USA	26	Mixed (mean age 37 ± 6 yrs)	Crossover RCT	Almonds (2 oz/day) vs pretzel	Resistance training	Not specified	8-week + 4-week washout time.	Creatine kinase, pain scale, torque test	Recovery, performance, soreness	Pre-run, post-run (3 days)	None reported

## 4 Data items

### 4.1 Primary outcome: muscle strength

Secondary outcomes: muscle mass, physical performance, and biochemical markers.

## 5 Critical appraisal of individual sources of evidence for muscle strength

### 5.1 Green gram

*Mudga* (green gram or *Vigna radiata* L.) is considered one of the most wholesome pulses due to its lightness, digestibility, and nourishing qualities. It supports *Mamsa dhatu* (muscle tissue) through its *Madhura rasa* (∼taste) and *Madhura vipaka* (∼post-digestive effect), which are nourishing and anabolic. These promote the formation and maintenance of healthy muscle tissue. Its *Laghu guna* (∼property) makes it easily digestible, which helps maintain optimal *Jatharagni* (∼digestive fire) and *Dhatvagni* (∼metabolic fire). *Mudga* is especially pacifying to *Kapha* and *Pitta dosha*s, thus supporting healthy muscle function without inducing inflammation [[15], [16], [17], [18]]. In this eight-week randomized controlled experiment, the effects of daily 18 g/day mung bean protein supplementation on lean body mass (LBM) and muscle strength in underactive vegetarian and vegan individuals were assessed. Of the 37 subjects that were enrolled, 25 (11 in the protein group and 14 in the control group) completed the study. While no significant differences in LBM or absolute strength measures were found between groups, the protein group showed a significant improvement in grip, knee flexor, and extensor strength (+2.9 %) compared to a decline in the control group. Additionally, LBM positively correlated with strength and protein intake^.^ [19]

### 5.2 A mixture of almonds, dried grapes, and dried cranberries

*Draksha* (dried grape or *Vitis vinifera* L.) nourishes and builds the *Mamsa dhatu* (∼muscle tissue) due to its *Madhura rasa* and *Snigdha guna*, which are *Brimhana* (∼bulk-promoting) in nature and promote tissue growth and rejuvenation. It enhances physical strength, stamina, and endurance by replenishing vital energy and supporting muscle tone, indicating *the Balya* (∼strengthening) property. It acts as a *Rasayana* (∼rejuvenation), promoting longevity, vitality, and tissue recovery. This rejuvenating effect helps in muscle repair, especially after fatigue or injury [[20], [21], [22], [23]]. This protocol describes a single-blind, randomized controlled trial designed to evaluate the effect of consuming almond, dry grape, and dried cranberry (AGC) on the psychomotor speed, exercise recovery, and endurance performance of male triathletes and cyclists who have received training. 96 participants will participate in an organized endurance training program that consists of a heavy training phase followed by a taper period, during which they will take either an AGC mix or an isocaloric oat-based control snack per day for four weeks. The primary outcome is endurance performance, which is assessed using a 5-min time trial. The AGC mix, rich in polyphenols, l-arginine, nitrates, and antioxidants, is hypothesized to enhance NO synthesis, reduce exercise-induced muscle damage, and preserve cognitive function under fatigue [24].

### 5.3 Turmeric

*Haridra* (*Curcuma longa* L., or turmeric) reduces *Shotha* (∼inflammation) through its *Ushna virya* (∼hot potency) and *Tikta* (∼bitter) and *Katu* (∼pungent) *rasa* (∼taste), which pacify *Ama* (∼toxins) and *Kapha dosha,* enabling better muscle recovery and performance. As a *Rasayana*, *Haridra* promotes the *Dhatu poshana* (∼tissue nourishment) process, especially the *Mamsa dhatu* (∼muscle tissue) [25,26]. A randomized, double-blind, placebo-controlled trial by Salehi et al. (2021) examined 80 healthy, moderately active young women who received 500 mg of curcumin daily for 8 weeks. The results demonstrated significant improvements in VO_2_ max (indicating enhanced aerobic capacity) and reductions in serum levels of lactate dehydrogenase (LDH), malondialdehyde (MDA), and C-reactive protein (CRP), suggesting lowered muscle damage and oxidative stress. However, body composition metrics such as weight, BMI, and lean body mass showed no significant changes [27]. The study explored the role of turmeric's active compounds on physical endurance and recovery. The study highlighted curcumin's potential to attenuate fatigue and enhance performance in young athletes, primarily by modulating inflammatory responses and supporting muscle tissue repair. These findings further strengthen the evidence base that turmeric supplementation may support muscle strength and recovery by reducing inflammation and oxidative stress [28].

### 5.4 Cow's milk

*Godugdha* (∼cow's milk) is ideal for *Brimhana* therapy, which promotes nourishment and growth of *Mamsa dhatu*. *Godugdha* indirectly improves muscle resilience, endurance, and muscle tone by enhancing *Ojas*. Acting as *Rasayana, Godugdha* is also used in chronic fatigue and emaciation. It is also ideal for *Santarpana chikitsa,* used in individuals suffering from *Dhatu kshaya* (∼tissue depletion) [[29], [30], [31]]. A randomized controlled trial by studied 35 elderly sarcopenic individuals across three groups (control, milk, and soy milk), all of whom underwent resistance training for 12 weeks. Significant improvements in handgrip strength were observed in the milk and soy milk groups, while calf circumference increased in the soy milk and control groups. The findings indicate that cow's milk, in combination with exercise, may support enhanced muscle strength in older adults [32]. In addition, a pilot study assessed the feasibility of a milk-based nutritional and resistance exercise intervention (MIlkMAN) in community-dwelling older adults. The intervention was well-accepted and led to favorable changes in physical performance, suggesting milk as a practical dietary component to support muscle function. The integration of milk intake with exercise may thus represent an effective, accessible strategy to counteract sarcopenia and age-related muscle loss [33].

### 5.5 Finger millet

Ragi (∼finger millet) is *Guru* (∼heavy), *Snigdha* (∼unctuous), and *Madhura kashaya rasa* (∼sweet–astringent taste). These properties support *Brimhana* action and *Vata dosha* pacification, helping in tissue building, especially *Mamsa dhatu*. In accordance with its Ayurvedic ability to nourish *Asthi* (∼bone) and *Mamsa dhatu*, ragi is high in calcium, iron, and amino acids [34]. This randomized controlled study investigated the effects of a 3-month intervention that combined physical activity with ragi laddu, a finger millet-based supplement, on biochemical markers and bone mineral density (BMD) in premenopausal women in Tamil Nadu, India, who were between the ages of 30 and 40. Out of the 720 women who underwent screening, 150 were randomly assigned to the control and experimental groups because they had low serum calcium and BMD. The experimental group engaged in resistance and strengthening exercises five days a week and consumed finger millet laddus three times per week. When compared to controls, the experimental group's blood calcium, phosphorus, alkaline phosphatase (ALP), bone mineral density (BMD), and physical activity scores significantly improved after the intervention. The study highlights that targeted physical activity and calcium-rich natural supplementation can effectively enhance bone health and prevent early osteoporosis in premenopausal women [35].

### 5.6 Spinach

The *Madhura vipaka* of *Palankya shaka* (∼spinach vegetable) indicates a nourishing and strengthening quality. The *Shita virya*, which prevents muscle inflammation, cramps, and excessive heat, is commonly associated with *Pitta dosha* imbalance [36]. *Laghu* (∼light) and *Ruksha* (∼dry) *guna* (properties) help to reduce *Kapha dosha* and prevent sluggish metabolism, supporting lean muscle development without fat accumulation. In this 12-week randomized, double-blind, placebo-controlled trial, the effect of moderate-intensity strength training (3 sessions per week) and daily supplementation with spinach extract (2 g/day) on muscle health in persons over 50 was evaluated. 45 participants were randomized to either spinach extract or a placebo. Although there were improvements in muscle mass and strength in both groups, the spinach group showed significantly greater increases in lower limb muscle mass, muscle quality, and isokinetic and isometric strength. The findings suggest that spinach extract supplementation, alongside resistance exercise, may enhance muscle fitness and support healthy aging [37].

### 5.7 Almonds

*Madhura rasa* and *vipaka, guru,* and *snigdha guna* of *Vatama* provide deep nourishment to *Mamsa dhatu*. Almonds are rich in essential fatty acids, proteins, and minerals, which aligns with Ayurvedic *Brimhana karma*, which nourishes and strengthens muscle fibers. It also strengthens the nervous system, which supports neuromuscular coordination and muscle performance. It also nourishes the brain and head region, which indicates its *Medhya* (∼nootropic) property [38,39]. In this randomized, crossover trial, 26 middle-aged, mildly overweight persons were tested for the effects of taking 2 oz of almond supplements daily vs. an isocaloric pretzel control on their ability to recover from exercise after a downhill run. After 8 weeks of dietary adaptation, participants completed a 30-min downhill run, followed by a 3-day recovery period. Almond consumption modestly reduced creatine kinase (CK) levels, improved muscle torque at 120°/s knee flexion, and decreased pain ratings during maximal contraction at 24–48 h post-exercise, compared to control. The findings suggest that almond supplementation may modestly enhance exercise recovery and support muscle performance after eccentric exercise [40].

## 6 Discussion

### 6.1 Summary of studies

Sarcopenia is a clinical condition with a multifactorial etiology from various biological mechanisms, including mitochondrial dysfunction, chronic inflammation, oxidative stress, and hormonal changes. Mitochondrial dysfunction leads to impaired energy metabolism and increased reactive oxygen species (ROS), contributing to muscle fatigue and atrophy. Chronic low-grade inflammation elevates cytokines (e.g., IL-6, TNF-α), promoting muscle breakdown. The oxidative stress causes damage to muscles and impairs regeneration due to age-related decline in antioxidant defenses. The hormonal changes, including reduced levels of testosterone, growth hormone, and IGF-1, weaken muscle synthesis and increase catabolism [41]. The Ayurvedic concept of *Mamsa Kshaya*, i.e., a depletion of muscle tissue, mirrors sarcopenic pathology.

This is one of the first scoping reviews regarding the impact of the *Ayush ahara* on muscle strength. The studies focused on muscle strength, muscle mass, exercise recovery, physical fitness, and biochemical parameters like oxidative stress, inflammation, etc. Body building foods are rich in protein, which helps build the tissues and muscles and shapes our body [42]. *Ayush ahara* may promote muscle tissue repair, particularly through protein-rich diets, but more biochemical studies are needed to confirm their mechanistic roles. The findings from nine rigorously selected randomized controlled trials provide compelling support for the *Brimhana*, *Balya,* and *Rasayana* qualities of several *Ayush Ahara*. The studies include combined dietary interventions with exercise training, enhancing muscle and bone health. The presence of co-interventions, particularly resistance or endurance exercises, in several included studies may have contributed to the observed improvements in muscle health outcomes. These co-interventions highlight the synergistic potential of traditional therapies with exercise-based approaches.

Green gram improves muscle strength despite minimal change in lean body mass, demonstrating the value of high-quality, plant-based protein in vegetarian populations. Almond and dried grape mix, which enhances exercise recovery and muscle torque, describes their antioxidant and NO-boosting effects. Curcumin from turmeric significantly lowered biomarkers of inflammation (CRP, LDH, MDA) and improved aerobic capacity, indicating its role in combating muscle inflammation and oxidative stress. Cow milk, rich in bioavailable proteins and micronutrients, improved grip strength and endurance when paired with resistance training, highlighting its role of *Santarpana chikitsa* in *Dhatu kshaya* conditions. Finger millet, through combined dietary and physical intervention, significantly improved bone mineral density and serum calcium, indicating its effectiveness in preserving musculoskeletal integrity. Spinach enhanced isometric and isokinetic muscle strength via nitrate and iron content, showcasing the synergy of nutrient-dense vegetables and physical activity. When consumed post-exercise, almonds led to better muscle recovery, confirming their *Balya* and *Medhya* properties as emphasized in Ayurveda. The mentioned studies are efficient because they minimize selection bias through randomized designs using double-blind methodologies.

A total of thirteen studies were excluded during the full-text assessment for the eligibility phase of this scoping review based on predefined inclusion criteria emphasizing original research on *Ayush ahara* and their role in muscle health or sarcopenia. Several studies focusing on foods not categorized under *Ayush ahara,* such as eggs [43] and soy milk [44], were excluded. Ayurveda regards *Ghrita* (∼cow's ghee) as nourishing in nature and supporting tissue development, including muscle health, through its *Rasayana* (∼rejuvenating) properties [45]. The studies, which show the potential role of *Panchatikta ghrita Ashwagandha* (*Withania somnifera*) supplementation, found significant improvements in muscle strength, muscle size, and recovery markers, suggesting its benefits in physical performance and muscle nourishment, but the studies were excluded from the final analysis as the intervention involved Ayurveda rather than *Ghrita* being assessed independently as a standalone dietary supplement [46,47].

Several trials investigated the effects of fortified milk products [48,49], milk protein [50], and milk fat globule membrane (MFGM), combined with exercise [[51], [52], [53], [54], [55]], which, while nutritionally relevant, did not align with the inclusion criterion of a traditional Indian dietary intervention aimed at enhancing muscle strength. Thus, despite their scientific merit, these studies were excluded to maintain methodological consistency. These excluded studies offer complementary insights that support the broader context of dietary impacts on muscle health and sarcopenia. Future research should focus on conducting larger, multi-center randomized controlled trials with more extended intervention periods to validate and expand upon the promising findings observed in these studies. Comparative effectiveness research is needed to determine the relative benefits of various dietary and exercise strategies, including different protein sources and types of physical training.

### 6.2 Limitations of the study

The limitation of the above studies was that they had a small sample size and a short duration that lasted only 4–12 weeks. The compliance issues vary because the diet and exercise interventions depend on participant adherence. These studies support personalized nutrition and exercise strategies targeting different age groups. The adequate nourishment of *Rasa* and *rakta* *dhatus* inherently supports *mamsa* *dhatu* development. Therefore, it is suggested that future reviews should also examine the effects of *Ayush ahara* on *rasa* and *rakta* *dhatus* to provide a more holistic understanding of their therapeutic potential. There was marked heterogeneity in populations, interventions, outcomes, and assessment methods of included studies. This diversity limited the ability to directly compare findings across studies and posed challenges in conducting a meta-analysis.

## 7 Conclusion

The healthy dietary pattern, including the *Ayush ahara,* could provide a personalized approach against sarcopenia due to the higher intake of millets (finger millet), pulses (green gram), vegetables (spinach), dry fruits (dried grapes & almonds), spices (turmeric), cow milk, and plant-based high protein. The reviewed evidence affirms that *Ayush ahara* positively impacts muscle strength, recovery, and inflammation, especially with physical activity. Integrating Ayurveda dietary interventions into modern nutritional guidelines could provide a personalized and sustainable approach to healthy aging. This scoping review concludes that *Ayush ahara plays a* role as a non-pharmacological approach for sarcopenia prevention but requires large sample studies to confirm the efficacy.

## Data statement

The data utilized for this review can be made available by the corresponding author upon reasonable request.

## Author contributions

RK: Conceptualization, Methodology, Investigation, Formal Analysis, Data Curation, Software, Writing - Original Draft, Writing - Review and Editing, Visualization, Supervision. UR: Formal Analysis, Data Curation, Software, Writing - Original Draft, Writing—Review and Editing, Visualization. SM: Formal Analysis, Data Curation, Visualization. MP: Conceptualization, Formal Analysis, Visualization, Supervision.

## Declaration of generative AI in scientific writing

None.

## Funding sources

None.

## Conflict of interest

The authors declare that they have no known competing financial interests or personal relationships that could have appeared to influence the work reported in this paper.
