# Supplementary material for: Efficacy of traditional Indian diet (Ayush ahara) on muscle strength and Sarcopenia: A scoping review
Source: J Ayurveda Integr Med. 2026 Jan 30;17(1):101265. doi: 10.1016/j.jaim.2025.101265 (PMC12874578; doi:10.1016/j.jaim.2025.101265)
Supplement: Multimedia component 1 [file mmc1.docx]

**Search Strategy in various databases:**

| **Database** | **PubMed** | **Total search results** |
| --- | --- | --- |
| #1 (Conventional science) | ("Muscle Strength" [Mesh] OR "Muscle Power" OR "Muscle Performance" OR "Physical Fitness" [Mesh] OR "Body Composition" [Mesh] OR "Sarcopenia" [Mesh] OR "Physical Performance") | 173474 |
| #2 (Ayurveda) | (Ayurveda [Mesh] OR "Ayurvedic Diet" OR Ayush Ahara OR "Traditional Indian Diet" OR "Vigna radiata" OR "Vitis vinifera" OR "Curcuma longa" OR "Eleusine coracana" OR "Prunus amygdalus" OR "Green gram" OR "Grapes" OR "Turmeric" OR "Finger millet" OR "Almond" OR "Cow's milk" OR "Milk" OR "Ghrita" OR " Cow's ghee" O "Drum stick" OR "Soybean" OR "Egg" OR "Herbal Supplement" OR "Traditional Dietary Supplement") | 169641 |
| #1 AND #2 | (("Muscle Strength" [Mesh] OR "Muscle Power" OR "Muscle Performance" OR "Physical Fitness" [Mesh] OR "Body Composition" [Mesh] OR "Sarcopenia" [Mesh] OR "Physical Performance")) AND ((Ayurveda [Mesh] OR "Ayurvedic Diet" OR Ayush Ahara OR "Traditional Indian Diet" OR "Vigna radiata" OR "Vitis vinifera" OR "Curcuma longa" OR "Eleusine coracana" OR "Prunus amygdalus" OR "Green gram" OR "Grapes" OR "Turmeric" OR "Finger millet" OR "Almond" OR "Cow's milk" OR "Milk" OR "Ghrita" OR " Cow's ghee" OR "Drum stick" OR "Soybean" OR "Egg" OR "Herbal Supplement" OR "Traditional Dietary Supplement")) | 927 |
| **Database** | **Scopus** | **Total search results** |
| #1 (Conventional science) | TITLE-ABS-KEY ( Muscle Strength OR Muscle Power OR Muscle Performance OR Physical Fitness OR Body Composition OR Sarcopenia OR Physical Performance ) | 23384 |
| #2 (Ayurveda) | ( TITLE-ABS-KEY ( Ayurveda ) OR TITLE-ABS-KEY ( Ayurvedic Diet ) OR TITLE-ABS-KEY ( Ayush Ahara ) OR TITLE-ABS-KEY ( Traditional Indian Diet ) OR TITLE-ABS-KEY ( Vigna radiata ) OR TITLE-ABS-KEY ( Vitis vinifera ) OR TITLE-ABS-KEY ( Curcuma longa ) OR TITLE-ABS-KEY ( Eleusine coracana ) OR TITLE-ABS-KEY ( Prunus amygdalus ) OR TITLE-ABS-KEY ( green gram ) OR TITLE-ABS-KEY ( grape ) OR TITLE-ABS-KEY ( Turmeric ) OR TITLE-ABS-KEY ( Finger millet ) OR TITLE-ABS-KEY ( Almond ) OR TITLE-ABS-KEY ( Cow milk ) OR TITLE-ABS-KEY ( milk ) OR TITLE-ABS-KEY ( Ghrita ) OR TITLE-ABS-KEY ( cow ghee ) OR TITLE-ABS-KEY ( Drum stick ) OR TITLE-ABS-KEY ( soybean ) OR TITLE-ABS-KEY ( egg ) OR TITLE-ABS-KEY ( Herbal Supplement ) OR TITLE-ABS-KEY ( Traditional Dietary Supplement ) ) | 941868 |
| #1 AND #2 | ( TITLE-ABS-KEY ( Muscle Strength OR Muscle Power OR Muscle Performance OR Physical Fitness OR Body Composition OR Sarcopenia OR Physical Performance ) ) AND ( ( TITLE-ABS-KEY ( Ayurveda ) OR TITLE-ABS-KEY ( Ayurvedic Diet ) OR TITLE-ABS-KEY ( Ayush Ahara ) OR TITLE-ABS-KEY ( Traditional Indian Diet ) OR TITLE-ABS-KEY ( Vigna radiata ) OR TITLE-ABS-KEY ( Vitis vinifera ) OR TITLE-ABS-KEY ( Curcuma longa ) OR TITLE-ABS-KEY ( Eleusine coracana ) OR TITLE-ABS-KEY ( Prunus amygdalus ) OR TITLE-ABS-KEY ( green gram ) OR TITLE-ABS-KEY ( grape ) OR TITLE-ABS-KEY ( Turmeric ) OR TITLE-ABS-KEY ( Finger millet ) OR TITLE-ABS-KEY ( Almond ) OR TITLE-ABS-KEY ( Cow milk ) OR TITLE-ABS-KEY ( milk ) OR TITLE-ABS-KEY ( Ghrita ) OR TITLE-ABS-KEY ( cow ghee ) OR TITLE-ABS-KEY ( Drum stick ) OR TITLE-ABS-KEY ( soybean ) OR TITLE-ABS-KEY ( egg ) OR TITLE-ABS-KEY ( Herbal Supplement ) OR TITLE-ABS-KEY ( Traditional Dietary Supplement ) ) ) | 795 |
| **Database** | **Embase** | **Total search results** |
| #1 (Conventional science) | 'Muscle strength'/exp OR 'Muscle strength' OR 'Muscle Power'/exp OR 'Muscle Power' OR 'Muscle Performance'/exp OR 'Muscle Performance' OR 'Physical Fitness'/exp OR 'Physical Fitness' OR 'Body Composition'/exp OR 'Body Composition' OR 'Sarcopenia'/exp OR 'Sarcopenia' OR 'Physical Performance'/exp OR ' Physical Performance' | 240544 |
| #2 (Ayurveda) | 'Ayurveda'/exp OR 'Ayurveda' OR 'Ayurvedic Diet'/exp OR 'Ayurvedic Diet' OR 'Ayush Ahara'/exp OR 'Ayush Ahara' OR 'Traditional Indian Diet'/exp OR 'Traditional Indian Diet' OR 'Vigna radiata'/exp OR 'Vigna radiata' OR 'Vitis vinifera'/exp OR 'Vitis vinifera' OR 'Curcuma longa'/exp OR 'Curcuma longa' OR 'Eleusine coracana'/exp OR 'Eleusine coracana' OR 'Prunus amygdalus'/exp OR 'Prunus amygdalus' OR 'Green gram'/exp OR 'Green gram' OR 'Grapes'/exp OR 'Grapes' OR 'Turmeric'/exp OR 'Turmeric' OR 'Finger millet'/exp OR 'Finger millet' OR 'Almond'/exp OR 'Almond' OR 'Cow milk'/exp OR 'Cow milk' OR 'Milk'/exp OR 'Milk' OR 'Ghrita'/exp OR 'Ghrita' OR 'Cow ghee'/exp OR 'Cow ghee' OR 'Drum stick'/exp OR 'Drum stick' OR 'Soybean'/exp OR 'Soybean' OR 'Egg'/exp OR 'Egg' OR 'Herbal Supplement'/exp OR 'Herbal Supplement' OR 'Traditional Dietary Supplement'/exp OR 'Traditional Dietary Supplement' | 500058 |
| #1 AND #2 | 'Muscle strength'/exp OR 'Muscle strength' OR 'Muscle Power'/exp OR 'Muscle Power' OR 'Muscle Performance'/exp OR 'Muscle Performance' OR 'Physical Fitness'/exp OR 'Physical Fitness' OR 'Body Composition'/exp OR 'Body Composition' OR 'Sarcopenia'/exp OR 'Sarcopenia' OR 'Physical Performance'/exp OR ' Physical Performance' AND 'Ayurveda'/exp OR 'Ayurveda' OR 'Ayurvedic Diet'/exp OR 'Ayurvedic Diet' OR 'Ayush Ahara'/exp OR 'Ayush Ahara' OR 'Traditional Indian Diet'/exp OR 'Traditional Indian Diet' OR 'Vigna radiata'/exp OR 'Vigna radiata' OR 'Vitis vinifera'/exp OR 'Vitis vinifera' OR 'Curcuma longa'/exp OR 'Curcuma longa' OR 'Eleusine coracana'/exp OR 'Eleusine coracana' OR 'Prunus amygdalus'/exp OR 'Prunus amygdalus' OR 'Green gram'/exp OR 'Green gram' OR 'Grapes'/exp OR 'Grapes' OR 'Turmeric'/exp OR 'Turmeric' OR 'Finger millet'/exp OR 'Finger millet' OR 'Almond'/exp OR 'Almond' OR 'Cow milk'/exp OR 'Cow milk' OR 'Milk'/exp OR 'Milk' OR 'Ghrita'/exp OR 'Ghrita' OR 'Cow ghee'/exp OR 'Cow ghee' OR 'Drum stick'/exp OR 'Drum stick' OR 'Soybean'/exp OR 'Soybean' OR 'Egg'/exp OR 'Egg' OR 'Herbal Supplement'/exp OR 'Herbal Supplement' OR 'Traditional Dietary Supplement'/exp OR 'Traditional Dietary Supplement' | 1326 |

***
